# Data Desa Presisi: A new method of rural data collection

**DOI:** 10.1016/j.mex.2022.101868

**Published:** 2022-09-20

**Authors:** Sofyan Sjaf, Sampean, Ahmad Aulia Arsyad, La Elson, Afan Ray Mahardika, Lukman Hakim, Sri Anom Amongjati, Rajib Gandi, Zessy Ardinal Barlan, I Made Godya Aditya, Sayyid Al Bahr Maulana, Muhammad Rifky Rangkuti

**Affiliations:** a Department of Communication and Community Development Sciences, Faculty of Human Ecology, IPB University, Indonesia; b College of Vocational Studies, IPB University, Indonesia; c Center for Agricultural and Rural Development Studies, IPB University, Indonesia; d Agribusiness Study Program, Faculty of Agriculture, Pembangunan Nasional Veteran University Yogyakarta, Indonesia

**Keywords:** Data Desa Presisi (DDP), Village data collection, Village development policy

## Abstract

Pseudo-development in rural areas often occurs due to the lack of availability of accurate data, in addition to the closed space for citizen participation. Based on this condition, we identify and evaluate various methods of collecting rural data in Indonesia as the basis for formulating development policies and programs. From the results of the identification and evaluation, we conclude that a new method in rural data collection is needed, called Data Desa Presisi (DDP). DDP is a village data collection method that synthesizes a census, spatial and community participation approach. This method puts the unit of analysis of the family and the individual in the Neighborhood Association (Rukun Warga-RW) as the smallest regional unit in the rural area. The presence of DDP is expected to help villages to plan, implement, monitor, and evaluate village development based on accurate data.

• We identified the village data collection methods used so far for planning and measuring village development.
• DDP is used for precise planning, implementation, monitoring-evaluation, and measurement of village development.
• This method can be used as basic village data because it is able to show development subjects with precision, namely: by name, by address and by coordinates.

## Graphical abstract

**Figure:**
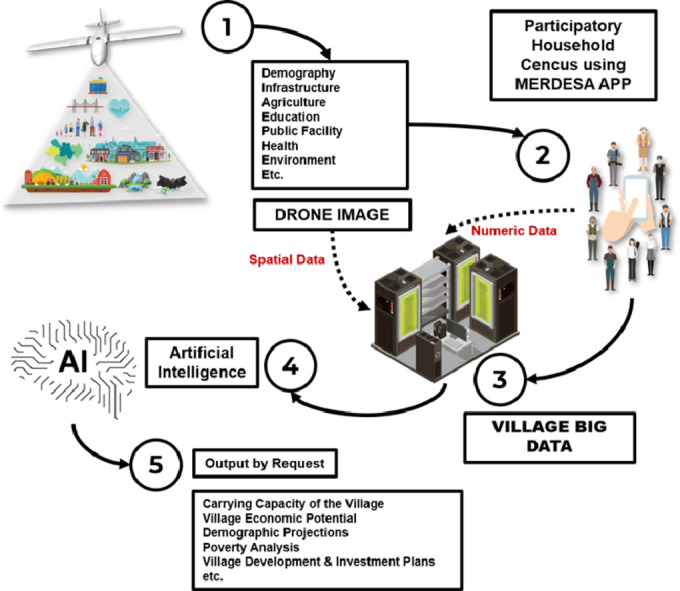
DDP Implementation Stages.

Specification Table

**Table:** 

Subject Area:	Social Sciences
More specific subject area:	Rural development and data collection
Method name:	Data Desa Presisi (DDP)
Name and reference of original method:	*Not applicable*
Resource availability:	*Not applicable*

## Method details

The availability of accurate data is very important for planning and measuring the success of development programs [1]. Without precise data, various development problems occur and result in pseudo-development which results in not achieving development goals [2]. The data inaccuracy is caused by the institution that has the authority related to data collection using only one method, quantitative or qualitative [3], [4], [5]. So far, village data in Indonesia has been obtained from the Village Potential (Podes) sourced from the Central Statistics Agency (BPS) [6] and Village and Urban Village Profiles (Prodeskel) sourced from the Ministry of Home Affairs [7]. The two methods of collecting village data were obtained from village officials (village head/village secretary/head of data affairs) using a paper-based questionnaire instrument [8]. By using this method, the village data presented is only in the form of aggregate data, not data in family units and individuals in the Rukun Warga (RW). As a result, village development policies are not right on target [7]. The Data Desa Presisi Method (DDP) presents basic data in the form of numerical and spatial data that is carried out by villagers using digital technology. DDP provides information on the current condition of the village related to 5 aspects of people's welfare, including: clothing, food and housing; education and culture; health, employment and social security; social life, protection of law and human rights; and infrastructure and the environment. Of the five aspects of people's welfare, 209 indicators are in the form of numerical data and 52 indicators are in the form of spatial data [1]. During data collection, we (university institutions) act as facilitators who carry out knowledge transfer in the form of capacity building (DDP approaches, techniques and analysis). This capacity strengthening is aimed at government officials and village residents who receive a mandate (decree) from the village head. With the possession of DDP by the village, the planning, implementation, monitoring and evaluation of village development will be right on target and according to the specified time target.

## New methods in rural data collection

### Village data inaccuracies and their causes

Various development measurements are produced by the government, such as: Gini Ratio Index/GRI, Human Development Index (HDI), Village Development Index (VDI), Developing Village Index (DVI), and Youth Development Index (YDI) [9], [10], [11], [12], [13]. The inaccuracy of measuring development achievements is due to the collection of basic data that is not participatory and is collected only based on the recognition of village government officials [8], [9], [10], [11], [12], [13], [14]. The inaccuracy of the Indonesian Government's basic data is shown from the Village Potential Data (Podes) in 2018 about 10.4% of unfilled questions from 849 questions and *Profil Desa dan Kelurahan* (Prodeskel) data in 2020 around 62-65% of unfilled questions from 939 questions [15]. The same thing was found that the inaccuracy of data obtained from the results of the National Sample Survey Office (NSSO) census of the Indian government, the level of gender inequality does not represent the actual condition of rural areas in India. [16].

In the context of the Indonesian case, GRI can only be calculated at the district level with available data. This has an impact on the inability to measure GRI at the village level. For example, North Tapanuli Regency's latest GRI score of 0.28 is only available in 2018 [17]. This GRI score is not able to show the contribution of the GRI score in every village in North Tapanuli Regency. By using DDP, the contribution of GRI at the village level can be known, for example the GRI in Sibandang Village, North Tapanuli Regency is 0.39 [18]. These measurements aim to see the achievement of development programs that prosper the community and educate the nation's life [2], [3], [4], [5], [6], [7], [8], [9], [10], [11], [12], [13], [14], [15], [16], [17], [18], [19]–22]. However, the measurement of development will never reach its goal, if the data used as a reference for calculations is not accurate. As a result, pseudo development will continue and have an impact on development failure [13].

In the context of development in Indonesia, rural areas play an important role as a representation of citizens' lives or subjects of development [21,22]. Therefore, accurate data is very important and determines the future of rural areas, as well as the realization of development goals. Thus, data accuracy plays an important role in decision making, policies and development programs [8], [9], [10], [11], [12], [13], [14], [15], [16], [17], [18], [19].

Until now, planning and measuring rural development in Indonesia uses the Village Potential (Podes) database sourced from BPS referring to the rules of Law Number 16 of 1997 concerning Statistics, Regulation of the Head of the Central Statistics Agency Number 49 of 2018 (Perka BPS No. 49/2018) concerning Guidelines for Collecting Village Potential Data in 2018, Presidential Regulation Number 86 of 2007 (Perpres No. 86/2007) concerning the Central Statistics Agency, as well as Village and Urban Village Profiles (Prodeskel) are sourced from the Ministry of Home Affairs which refers to the Minister of Home Affairs Regulation Number 12 of 2007 (Permendagri No. 12/2007) concerning Management of Village and Urban Village Profile Data Collection [6], [7], [8]. Furthermore, these two data sources use a census approach with village government officials (village head/village secretary/head of village data affairs) as respondents.

In contrast to the Podes and Prodeskel approaches, DDP uses a digital-based census approach which is combined with a spatial approach, and places village residents (youth) as actors in collecting data in the village (enumerators). Then, in contrast to Podes and Prodeskel, DDP places the head of the family as respondents in data collection.

### The presence of a new methodology

The inaccuracy of the rural data collection system in Indonesia has prompted us to formulate DDP as a new method of collecting rural data. This method is an inclusive approach that places the relationship between humans and technology to collect rural data by considering the spatial dimension, digital technology, citizen participation and census [1,[2], [3], [4], [5], [6], [7], [8]. The use of this method is to explore various parameters that are categorized into five aspects of people's welfare, including: clothing, food and housing (63 parameters); education and culture (8 parameters); health, employment and social security (72 parameters); social life, legal protection and human rights (27 parameters); and infrastructure and environment (15 parameters). In addition, there are 24 parameters of family identity that explain the respondent's information [1].

### DDP implementation stages

**Fig. 1:**
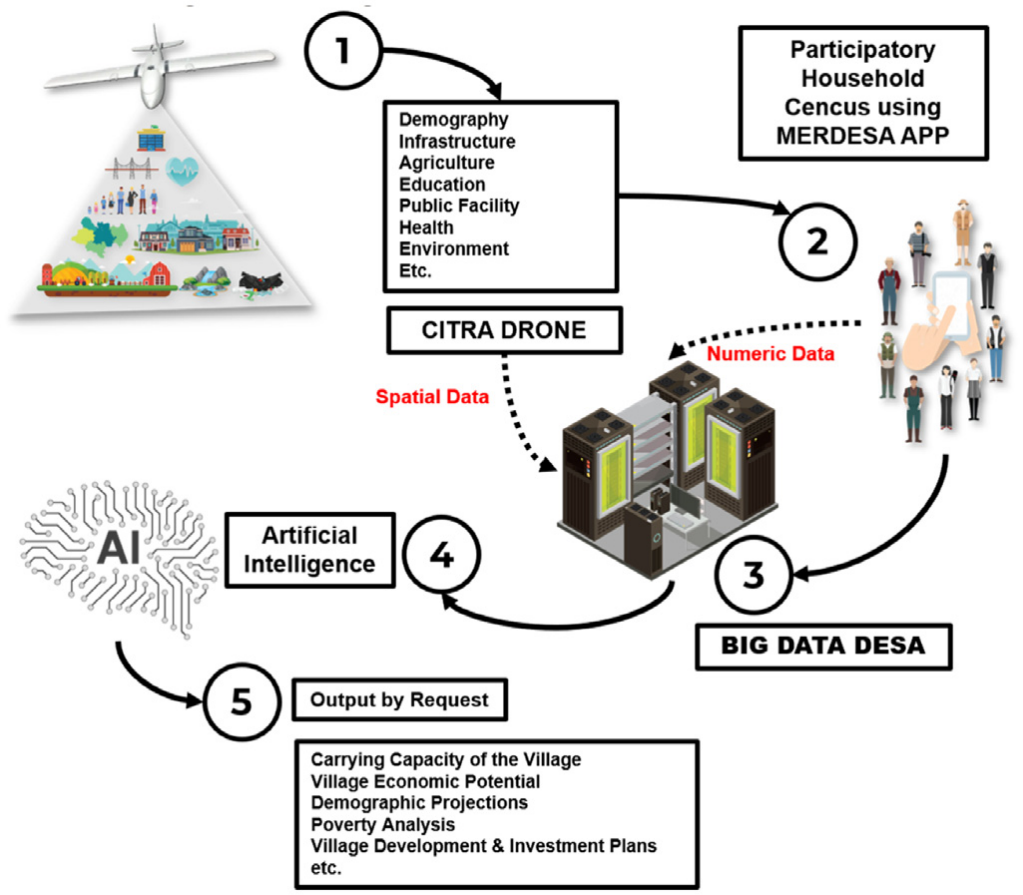
DDP implementation stages.

In general, the DDP implementation (practice) stage consists of five stages: (1) producing high-resolution images. The instruments used at this stage are drone technology to produce high-resolution images; (2) conduct a participatory-based household census. At this stage, the involvement of village youth is very important. Prior to data collection, village youth were recruited in each RW. Those who are recommended by the village government are trained to use the MERDESA Census application instrument that we created; (3) data storage (numeric and spatial). At this stage, all data (numeric and spatial) are stored on the server; (4) preparation of the village development size algorithm [1]. This stage is oriented to develop artificial intelligence for village development; and (5) developing digital applications to address village needs (Fig. 1).

Based on the five stages of DDP practice above, in general DDP is grouped into three activities: drone-based (spatial) mapping activities; digital-based participatory census activities; and activities for the preparation of artificial intelligence based on village needs. The descriptions of the three activities in question are as follows:

#### Drone-based mapping activities and satellite imagery (spatial)

This activity starts from preparing everything related to drone-based mapping and satellite imagery activities that are carried out in a participatory manner, such as: reviewing report documents, providing field survey tools and materials, and landscape/land units [23]. The preparation of the survey in this research aims to obtain an overview of the area as a whole through the collection of information from available/relevant data and maps, to be able to assist in the analysis and implementation of the survey in the field. Second, interpretation of landscapes/land units from DEM data and remote sensing images. Regional and spatial units are used as the basis for field planning and the preparation of village maps as study material to support the formation of DDP. Before conducting a survey, it is necessary to prepare materials and equipment so that the survey can run well. The equipment and materials used include:

• Map of activity locations (source: Geospatial Information Agency-BIG);
• Landsat satellite imagery (source: SasPlanet);
• Drone flight plan design map;
• Computers and Laptops equipped with spatial mapping support software such as ArcGIS Desktop, Global Mapper, Google Earth, and AgisoftPhotoscan;
• DJI Mavic 2 Pro Quad Copter Drone and its supporting equipment;
• Mobile Phone equipped with applications such as; DJI GO 4, Pix4Dcapture, DJI+Ctrl, Avenza Maps and MERDESA Maps; and
• Global Positioning System (GPS) Handle: GPSMap 64s Garmin.

#### Survey implementation

The implementation of the survey is a field data collection process which is the main series of spatial data collection activities in building DDP. The stages of collecting field data include:

1) Focus Group Discussion (FGD) with village officials and community;
2) Tracking and pinpointing village and RW boundaries with the community and local village officials and representatives of neighboring villages;
3) Aerial photography of the village area using drones;
4) Tracking and monitoring of public facilities and infrastructure in village areas;
5) Identification of biodiversity in tree, seedling and understorey categories;
6) FGD verification of village spatial data; and
7) RW-based work map creation.

#### Spatial data processing and analysis

Spatial data processing and analysis is the process of interpreting field survey data and other supporting data to display DDP geostatistically. The stages of processing and analyzing spatial data include:

1) Plotting data on village and RW boundaries, identification of facilities and infrastructure and village biodiversity;
2) Aerial photo mosaic;
3) Drone image correction;
4) Digitizing upright drone images and upright satellite images; and
5) Village map creation.

Spatial data analysis adapted to village conditions and needs, for example disaster analysis, village spatial planning, village natural resource potential, SDGs analysis, and others.

### Digital-based participatory census activities

#### Census and participatory approach

The census approach in constructing DDP is a follow-up to the spatial approach. The work map that becomes the output of the spatial approach is used as a guide in the census approach. This is an effort to minimize individuals or every person in the village that is missed to be recorded. Several stages are carried out by the census approach, namely: census and FGD preparation, census and FGD implementation and census data processing.

#### Census preparation and FGD

At the initial stage, the team conducted training for village youth representatives from the RW scope. Each RW recruits 3-5 village youths who will be involved in the census process. Training and capacity building are carried out by providing orientation on the importance of DDP as a basis for development planning, technical capacity building in applying MERDESA Census Application to conduct censuses, strengthening understanding in reading work maps in MERDESA Census Application, and increasing understanding of metadata/operational definitions of census parameters. These village youths will later be involved in the process of collecting census data for each household based on the address, name, and coordinates of each RW (Table 1).

**Table 1:** Differences in village data collection procedures and mechanisms for Podes, Prodeskel and DDP.

Difference		Rural data collection		
		Podes	Prodeskel	DDP
1.	Juridical norms	Law No. 16/1997, Perpres No. 86/2007, Perka BPS No. 49/2018	Permendagri No. 12/2007	There isn't any. Although there is an opportunity in Presidential Decree No. 39/2019.
2.	Data category	Location Description, General Information on Village/Urban, Population and Employment, Housing and Environment, Natural Disasters and Disaster Mitigation, Education and Health, Social Culture, Sports and Entertainment, Transportation, Communication and Information, Land Use, Economy, Security, Finance and Village Assets, Use of Village Funds, Community Development and Empowerment Village/Urban, Description of Village/Urban Government Officials,	Three aspects: (1) basic family data; (2) village potential; and (3) village development	5 aspects of welfare: (1) clothing, food, housing; (2) Education and culture; (3) health, employment and social security; (4) social life, protection of law and human rights; and (5) infrastructure and environment
3.	Approach	-	Collecting data from village officials.	Data collection using the Drone Participatory Mapping (DPM) approach
4.	Instrument	-	Paper-based and website-based questionnaires.	MERDESA census application (smartphone base)
5.	Respondents/informants	-	Village officials	Village officials, all families living in the village
6.	Data type	-	Numerical	Numerical and spatial
7.	Citizen participation	-	None	Head of RW, head of RT, community leaders, village youth, NCO for Public Order and Security (Babinkamtibmas), and Village Trustee (Babinsa)
8.	Position of the village and residents	-	Object	Subject

Source: (Researcher).

In addition to preparing human resources for the implementation of the census, the research team developed coordination with the village authorities to prepare for the implementation of the FGD. At this stage, FGDs are prepared to explore or collect qualitative village data in a participatory manner. Qualitative data consists of local village history, seasonal calendar, social stratification, village economic potential, village institutions, and problem trees. The qualitative data collection process involves resource persons from community leaders to understand the historical and actual conditions of the village. The importance of collecting qualitative data to explore the collective memory of residents in understanding their village situation [24].

#### Implementation of the census and FGD

This stage is carried out by village youth (enumerators) representing each RW to record every person in the household (census) in the area of each RW. In the census process, enumerators are equipped with the MERDESA Census Application which can be accessed via the enumerator's smartphone. Each enumerator will visit each household by asking various questions about the status of the building, the identity of the respondent, data on land ownership, participation in village activities, ethnicity, consumption level, employment, side work, number of household members, age of household members, number of household members. family in the household, illness, accessibility to health insurance, sanitation, communication, living conditions, frequency of eating, food menu, cooking fuel, source of washing water, history of cultivated commodities, non-agricultural income, average family expenditure, as well as the coordinates of residents' houses which are automatically identified in the MERDESA Census Application. The description of the census parameters with the MERDESA Census Application is shown in Table 3.

**Table 2:** Census parameters with MERDESA census application.

Respondent target	Variable	Number of parameters (questions)	Information
Head and Family Members	Family Identity	24	Related the Identity of the Head of the Family, Family Identity
	Education and Culture	8	Related to Education, Ethnicity, Religion, Educational Status, and tuition fees
	Infrastructure and Environment	15	Condition of the house yard, Economic assets owned, Garbage disposal sites, ownership of Communication Equipment
	Social Life, Legal Protection, and Human Rights	27	Status of Residence, Social Security and Assistance Program, Monthly fees and expenses, Total vehicle assets, Organizational participation, Entertainment, religious fees and donations
	Health, Employment and Social Security	72	Employment, Social Security, Diseases, Health Programs, Access and Agricultural Land Commodities, Livestock Ownership
	Clothing, Food and Housing	67	clothing, housing, and food consumption

During the census process, the research team monitored and evaluated the data entered on the server, ensured that the inputted data was valid, and compiled the output of data analysis. The process of monitoring and evaluating census data is carried out by supervision in the MERDESA Census Application and regularly face-to-face meetings with village enumerators are held to ensure the census process runs well.

#### Methods validation

The integration of spatial data and numerical data in the DDP method begins with the use of work maps that are inputted into the MERDESA Census application (digital work maps). The digital work map functions as a social enumerator navigation in tracing every house and building in the village. This digital work map is spatial information on the distribution of settlements and other buildings in units of RW/Dusun/Environment in rural areas. The data displayed on the map includes: names of provinces, regencies/cities, districts, villages, village codes, codes of settlements and other buildings, and coordinates (longitude/latitude).

The technique of using digital work maps works if the social enumerator is at the coordinates of the code points for houses and other buildings that have been assigned pin-points. Spatial information from this data is the result of digitizing building parcels through geometrically corrected drone images. The basis for assigning codes to each building is the shape of the roof perpendicular to the earth's surface. With this technique, the total number of buildings identified as a whole based on the code. However, each of the identified codes for houses and other buildings cannot be precisely ascertained, whether the roof base used is in accordance with the number of buildings per unit or is it still a combination of units. As for validation to ensure this, through verification results from social enumerators who conduct censuses to ensure that each code is appropriate or not. Several possible results of the verification carried out, such as: one building code may combine more than one building. Or conversely, two or more existing building codes may actually be just one building.

Furthermore, the results of the social enumerator's verification of the initial work map made by the spatial team are re-validated to ensure that each building code given is in accordance with the actual conditions in the village. This validation confirms the identification of building codes that are inhabited by residents or not inhabited by residents. After verification and validation have been carried out, the information from the census results is presented geostatistically according to the required thematic categories. The process of integrating this spatial and numerical data is to produce a family-based DDP in every RW/Dusun/Environment in rural areas.

For the social data validation method, it is carried out through: first, a census based on a RW-based digital work map. As previously mentioned, the digital work map functions as a social enumerator navigation for conducting door-to-door censuses. This work map is also capable of verifying and validating non-digitized buildings and households; second, the recruitment of village youth as RW-based social enumerators. The purpose of recruiting RW-based social enumerators from village youth is to involve citizens as subjects who have an understanding of village spatial planning, social, economic and cultural conditions of rural communities, as well as the transformation of knowledge from universities to citizens; and third, intensive assistance by the census supervisor. Intensive assistance starts from the training process for social enumerators, organizing social enumerators, planning strategies for census completion, monitoring, evaluating, confirming invalid data during the census process, up to the preparation of DDP outputs.

For example, the application of the DDP method in Sibandang Village, Muara District, North Tapanuli Regency, North Sumatra Province. This 321.52 hectare village is inhabited by 294 families, with a total of 409 digitized buildings, consisting of 247 inhabited buildings and 162 uninhabited buildings. Spatial mapping activities were carried out by 2 people for 7 days, in the form of taking village images (using drones), tracking village/RW boundaries, pointing out facilities and infrastructure in villages, and making digital work maps. Meanwhile, the census activities were carried out by 20 enumerators as representatives from 3 Dusun/RW, and accompanied by 3 social supervisors. The census activity was carried out for a total of 5 days, with the first day for enumerator training and the last day for evaluation.

### Artificial intelligence development activities

#### Processing and preparation of development planning

In this paper, the implementation of DDP takes a case study in Sibandang Village, Muara District, North Tapanuli Regency, North Sumatra Province. The output of the spatial approach is in the form of a thematic map based on drone imagery and verified in a participatory manner by villagers. The output of the Census and participatory approach is in the form of a collection of data sheets containing information on buildings, family and individual identities, as well as data related to predetermined census parameters. The data is then processed and collected in the form of monographs as outputs that can be presented infographically in the form of base maps and thematic maps. Existing data sheets can be further analyzed for various purposes.

This data sheet is superior to the data collection method carried out by BPS because it is taken directly by the population, and there are procedures that require enumerators to actually collect data one by one based on names, addresses, and coordinates. Likewise, the map generated from the spatial approach is superior to the spatial data output from BIG because the determination of the boundaries of villages, RWs, and even RTs, is verified directly by villagers as statutes or rules that already exist in their community. These two advantages make DDP with an approach that integrates spatial, quantitative, and qualitative aspects reliable as a baseline for village development planning.

### Spatial and social data integration

DDP data collection produces integrated data between spatial data and numerical data. The data integration provides an overview of the actual condition of the village. The results of the analysis and implementation of DDP provide a portrait of the use of built and non-built land in the Rukun Warga (RW) analysis unit or community environment unit. In addition, the integration of spatial and social data is also illustrated from the results of the RW-based census which are displayed spatially in thematic form on each indicator of people's welfare. In Sibandang Village, it is presented in hamlet units because the hamlet is a social unit in society because it represents kinship and kinship ties [25]. Hamlet (Dusun) is a traditional institution under the village which is a meeting place for common interests or a cultural mediation space where cultural agroecology is seen as a representation of the agency and actions of farmers, the issue of participation in rural development can be seen more broadly [26].

**Fig. 2:**
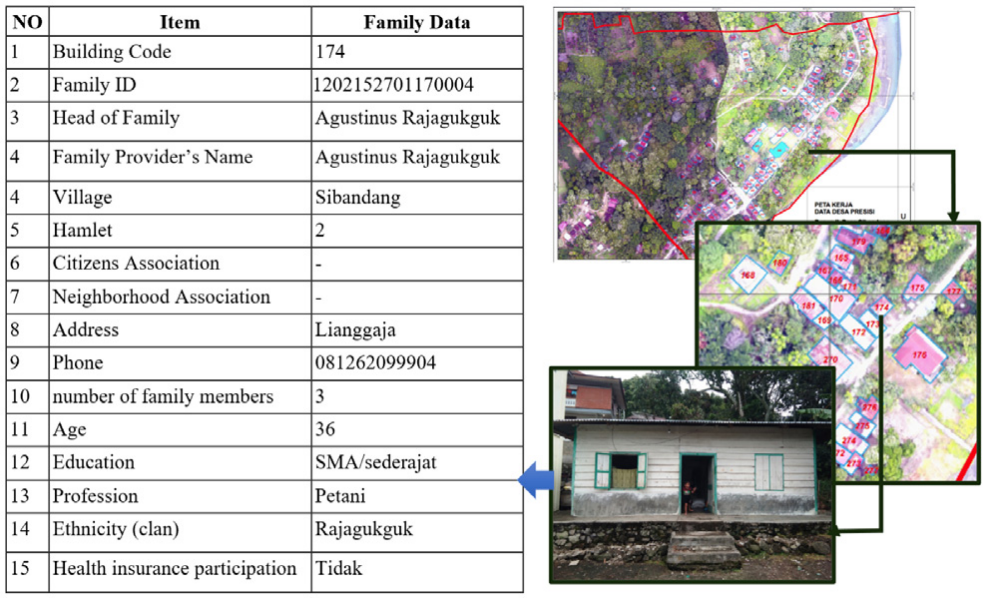
Demography aspects based on spatial and numerical data.

Another interesting thing is the implementation of precise village data collection in the population aspect. Data is presented not only in numerical form. But also, presented in a spatial form. The presentation process can be seen in Fig. 2, showing that all buildings or houses in the village were photographed properly, then coded, then identified numerically and the physical condition of the houses in the village can be known. The house code provides population information that can be presented in the DDP Webgis based on name, address, and coordinates.

**Fig. 3:**
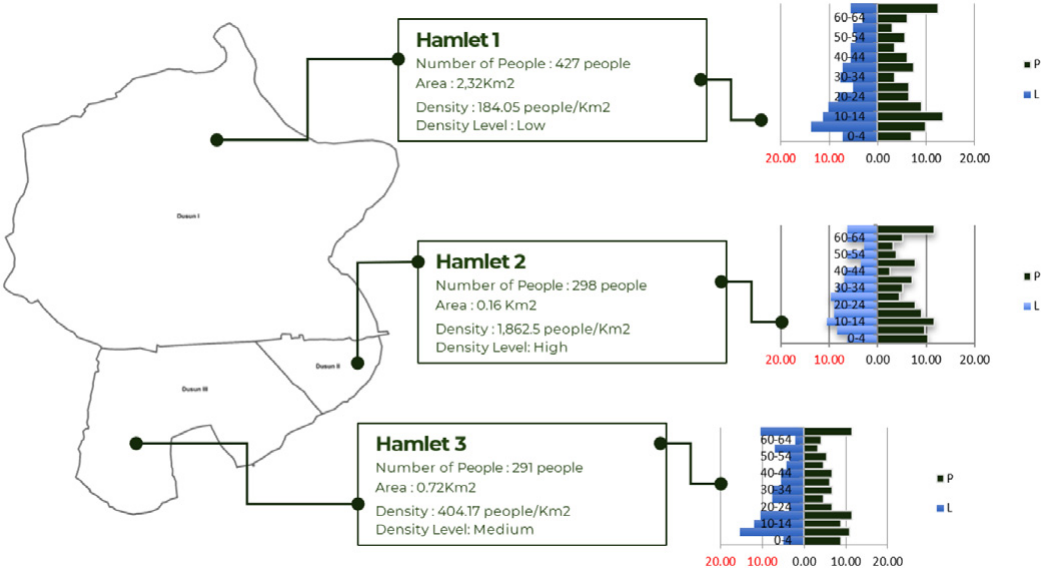
Population density distribution and the population pyramid of Sibandang Village (hamlet based).

By using DDP, the distribution of population density and population pyramid can be well presented, per RW or local community unit. Figure 3 provides information on population density in hamlet units. The population pyramid shows the distribution of the population by age and sex in each hamlet, Sibandang Village. This village demographic information can make it easier to plan development with a human development approach.

**Fig. 4:**
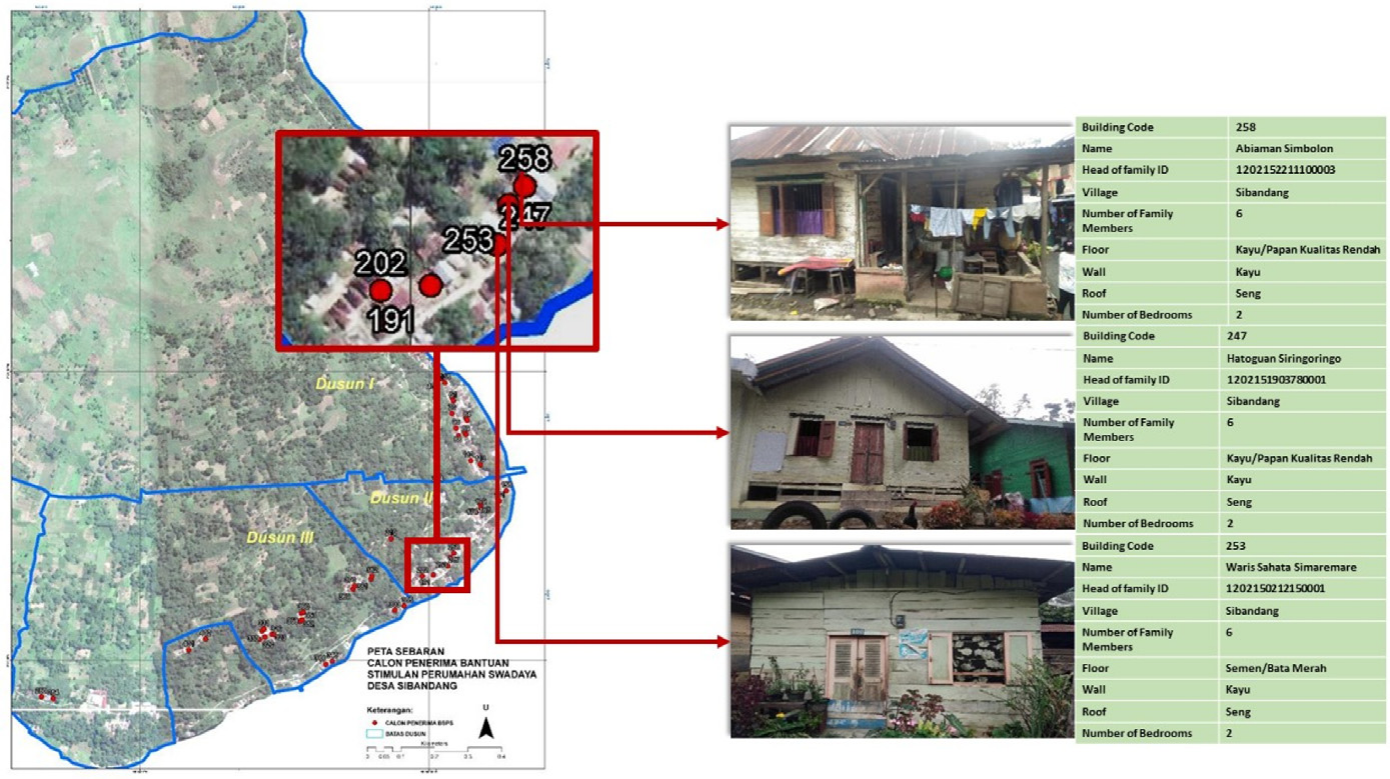
Distribution of uninhabitable houses in Sibandang Village.

Utilization of DDP can contribute to development policies, such as improvement of development policies based on handling household-based problems and needs. One of the contributions of DDP is to present information on uninhabitable houses that are displayed spatially. As shown in Fig. 4 which shows a map of the distribution of uninhabitable houses in Sibandang Village. Uninhabitable houses can be identified spatially, as well as well described. Related to the building code, the name of the owner of the house, the number of family members, what floors, roofs and walls are made of, and the number of bedrooms in the house.

**Fig. 5:**
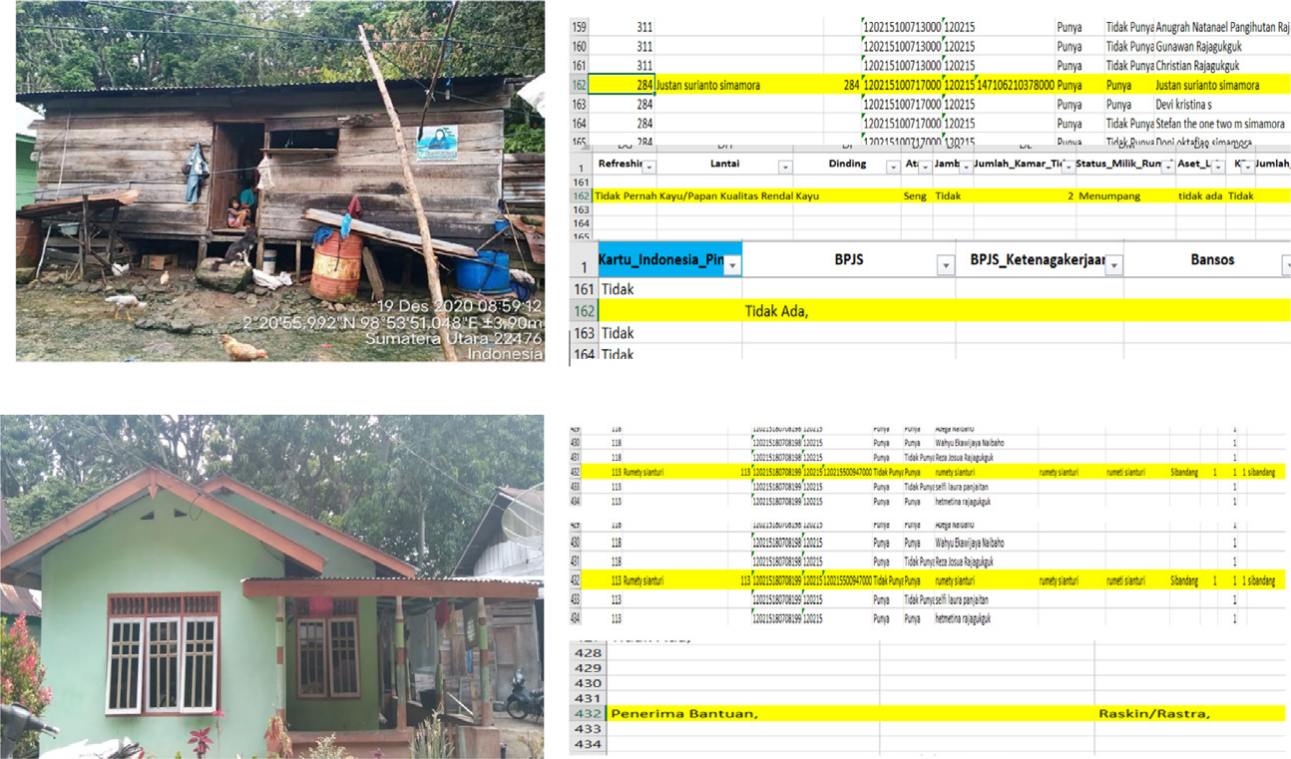
Prosperous families who receive Raskin/Rastra social assistance.

Still in the interest of development, DDP is also able to clarify and clearly describe which poor families do not receive social assistance (Bansos), and which families are not poor but receive Bansos. The following is data on poor families who do not receive social assistance, as seen from the physical condition of the house and numerical data. On the other hand, families who are classified as prosperous, but are recipients of social assistance. This can be seen in Fig. 5 seen through the display of the physical condition of the house and its numerical data.

### Calculating the quality of village SDGs

**Fig. 6:**
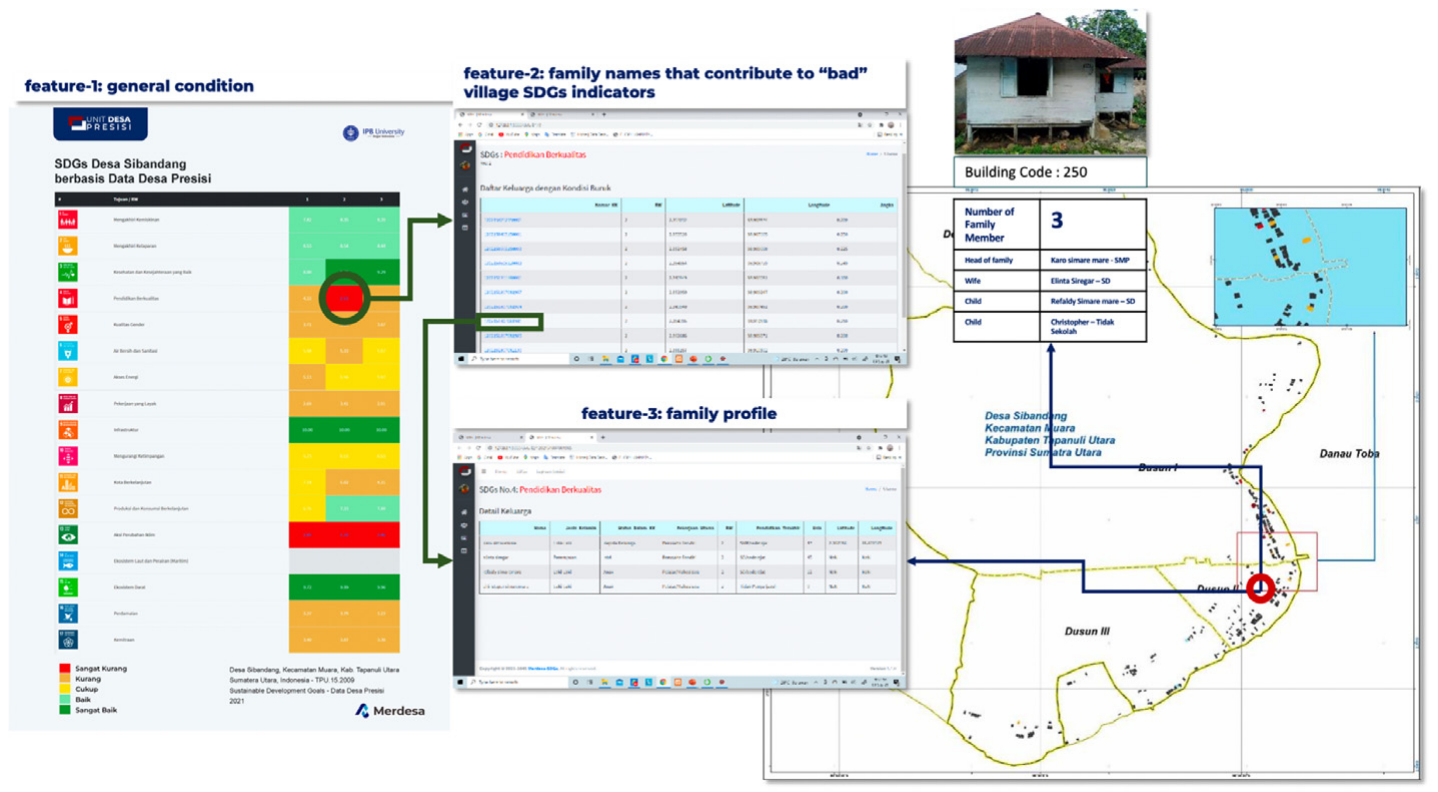
SDGs general condition and family names that contribute to “bad” village SDGs indicator.

Using precise village data, we can calculate the quality of the Village SDGs. In Fig. 6, the general condition of the SDGs in Sibandang Village is presented, related to their status which is categorized from very bad to very good, based on color presentation. The red color indicates the very poor status of the SDGs. Seen in SDGs point 4, the quality of education, in Hamlet (Dusun) 2 is red. This means that the quality of education is red (very poor) due to the large number of families in Dusun 2 Sibandang Village contributing to the poor quality of SDGs point 4. Not only showing the number of families, DDP can also show the complete profile of the family along with the position of the family coordinates on a spatial map as well as a photo of the house.

### “method-problem fit” map

We investigate study methods commonly used and accepted by the wider research community such as mapping which combines quantitative and qualitative approaches [3]. Research approaches in a variety of sciences continue to be developed by integrating a spatial approach in the unit of analysis. One of the research studies in the development of social analysis methodology is the calculation of state fragility based on the implementation of laws, policies, and development programs. The calculation approach combines quantitative and qualitative approaches [27]. This approach is more specifically continued by [28] by calculating the balance and economic shocks spatially with a quantitative spatial economic approach. Different research results are used in forest policy analysis that prioritizes a qualitative approach [29].

The results of previous studies rely on a partial approach in analyzing the implementation of policies, laws, and development programs. This deficiency is complemented by a holistic DDP approach that integrates the three approaches (spatial, census and participatory) to answer the basic problems faced by villagers. The data collected in the DDP is presented with “Problem-method fit” which is based on the identification and analysis of problems from spatial and census data. The DDP approach has been used in development policy themes and land use analysis.

The results of previous studies rely on a partial approach in analyzing the implementation of policies, laws, and development programs. This deficiency is complemented by a holistic DDP approach that integrates the three approaches (spatial, census and participatory) to answer the basic problems faced by villagers. The data collected in the DDP is presented with “Problem-method fit” which is based on the identification and analysis of problems from spatial and census data. The DDP approach has been used in development policy themes and land use analysis. So far, the results of the DDP have been used by the central government (Ministry of Social Affairs of the Republic of Indonesia) for the purpose of distributing social assistance for Uninhabitable Houses (Rutilahu). At the regional level, the Government of North Tapanuli Regency, North Sumatra Province has also adopted DDP for the purposes of updating village data and determining village boundaries. This is stated in the North Tapanuli Regent Regulation Number 53 of 2022 (Perbup No. 53/2022) regarding Precision Village Data as a form of local government recognition of DDP in quality regional development planning [30].

At the village level, Gelaranyar Village, Pagelaran District, Cianjur Regency, West Java Province has also used the DDP results to determine poor families in their villages. Apart from the government, universities use DDP for research purposes (thesis, theses, dissertations and scientific journals) and community service (farmers and village youth empowerment programs) [8], [9], [10], [11], [12], [13], [14], [15], [16], [17], [18], [19], [20], [21], [22], [23], [24], [25], [26], [27], [28], [29], [30], [31].

It is important to note that some of the previous research articles used only specific methods and were limited to certain cases. Moreover, in several papers we found it quite difficult to distinguish whether a method was specifically used for core themes, or contextual analysis, or both [32,33]. In such cases, we consider the method used for both. Furthermore, when we look for trends in popular methods (very often used), we complement and develop new methods in rural data collection systems as well as development policy analysis research approaches.

### The role of the responsible researcher in the selection and adaptation of methods

Rather than being an easy solution to any research problem, our findings provide guidance and show trends in only popular methods. Decisions about methods need to be critically examined and possibly supplemented by additional methods by the researcher. Researchers can follow the trends found in this paper and use the most frequently (popular) methods used quantitative methods with statistical analysis and qualitative methods with descriptive analysis. Then, a combination of quantitative and qualitative methods has become a trend in academic research approaches. The use of academic research methods in rural data collection systems has never been used rigorously. In practice DDP as a rural data collection system adopts academic research approaches, digital technology, artificial intelligence to formulate new methods. The formulation is suitable for analyzing empirical cases, needs of villagers, rural potential, research preparation, and development planning. However, this approach must be supported by quality research and must be measured for originality and novelty in each method development. DDP as a new method, must be able to present actual and contextual data in providing a new understanding of certain social phenomena. In fact, similar research should be conducted more frequently to provide new and limited original ideas.

In rural data collection systems in various countries, the possibility of giving birth to new innovation systems. This is because the nature of research and rural data collection methods in each country is constantly evolving. Researchers can try new methods that were rarely used until recently to find new insights. Therefore, we encourage researchers to critically and realistically consider innovation or adaptation of new methods in rural data collection systems as well as in research approaches that are more effective and efficient. Another consideration innovation in rural data collection systems should consider the use of the latest technology to address data problems. The use of this technology must be able to answer, describe, and present data on residents' problems in an actual and precise manner based on names, addresses, and coordinates.

## Conclusion

Data inaccuracies occur because the approaches, methods, and instruments used do not meet the rules of data collection and tend to be top-down, resulting in Pseudo Data. DDP that uses Precision Village Mapping, is designed through a synthesis of census, spatial, and participatory approaches that are able to produce data that has high accuracy and can be used as a basis for development planning. The advantage of DDP is that it places villagers as data subjects, displays a detailed village potential profile, and is able to ensure the accuracy of development measures. However, DDP requires a regulatory or policy basis that can be used as a reference for its implementation in the field.

DDP provides accurate geostatistical data and can be used as a basis for village development planning. There is a need for legality in the form of regulations or policies that protect and can be a reference by various institutions or agencies related to the implementation of DDP. The approaches and methods used in DDP can continue to be studied academically, involve multi-stakeholders and empower the community as data subjects. It is important to have an understanding of the methods of collecting and using integrated data, as well as having interoperability between agencies/institutions using data.

Considering the potential and challenges faced by the research team in formulating the rural data collection system method. The researchers mapped the country's rural data collection methodology as the basis for analyzing development policies, laws, and programs. The results of the mapping found that the state was trapped in the use of pseudo data because it denied citizen participation as the subject of data collection. These findings are used to improve and refine the rural data collection system that is practiced by the state. DDP as a rural data collection method is present as an answer to the problem of data inaccuracies and errors in measuring development achievements.

DDP is developed based on the use of specific approaches that are popular in various research themes. DDP can also be used as a quick assessment guide for researchers and policy makers in formulating specific strategies to address basic problems faced by citizens. for their studies more efficiently. However, researchers need to critically assess its suitability for their study. They are also encouraged to explore the possibility of using less standard methods as new innovations in research.

## Conflicts of Interest

The authors confirm that there are no conflicts of interest.

## References

[1] Sjaf S., Elson L., Hakim L., Godya I.M. (2020).

[2] Sjaf S., Kaswanto K., Hidayat N.K., Barlan Z.A., Elson L., Sampean S., Gunadi H.F.F. (2021). Measuring achievement of sustainable development goals in rural area: A case study of Sukamantri Village in Bogor District, West Java, Indonesia. Sodality.

[3] Creswell J.W. (2016). Kuantitatif, dan Campuran.

[4] Hargreaves J.R., Morison L.A., Gear J.S.S., Makhubele M.B., Porter J.D.H., Busza J., Watts C., Kim J.C., Pronyk P.M. (2007). Hearing the Voices of the Poor”: assigning poverty lines on the basis of local perceptions of poverty. A quantitative analysis of qualitative data from participatory wealth ranking in rural South Africa. World Dev..

[5] Howe G., McKay A. (2007). Combining quantitative and qualitative methods in assessing chronic poverty: the case of Rwanda. World Dev..

[6] BPS, Village Potential Statistics of Indonesia 2021 (Jakarta, 2021).

[7] Kemendagri, Buku Panduan Sistem Informasi Profil Desa dan Kelurahan (Jakarta: Direktorat Jenderal Pemberdayaan Masyarakat dan Desa Kementerian Dalam Negeri, Republik Indonesia, 2012).

[8] Pitaloka R.D., Hendriyani H., Eriyanto E., Haryatmoko H. (2022). Communication practice in village data collection. J. Stud. Komun. (Indonesian J. Commun. Stud.).

[9] K. Ruslan, Memperbaiki Data Pangan Indonesia Lewat Metode Kerangka Sampel Area (Jakarta, 2019).

[10] Chambers R., Kakwani N., Silber J. (2013). Many Dimensions of Poverty.

[11] Chambers R., Ehrenpreis D. (2006). What is Poverty? Concepts and Measures.

[12] R. Chambers, Poverty and livelihoods: whose reality counts? (1995).

[13] Chambers R. (2008).

[14] Wijoyono E. (2021). The utilization of village-information system for integrated social welfare data management: actor-network theory approach in Gunungkidul regency. J. Tek..

[15] Pitaloka R.D. (2022).

[16] K. Mehta, Estimates of Women's Labour Force Participation: Rectifying Persisting Inaccuracies (2021). doi:10.13140/RG.2.2.18315.82729.

[17] (2021).

[18] Sjaf S. (2021). Covid 19, Ketimpangan, Kemiskinan, dan Pengangguran Di Pedesaan. Kompas.

[19] Sjaf S. (2019).

[20] Sjaf S. (2017). Merebut masa depan pertanian. Kompas.

[21] Sampean, Sjaf S. (2020). The reconstruction of ethnodevelopment in Indonesia: a new paradigm of village development in the ammatoa kajang indigeneous community, Bulukumba Regency, South Sulawesi. Masyarakat.

[22] Sampean E., Wahyuni S., Sjaf S. (2019). The paradox of recognition principles in village law in Ammatoa Kajang Indigenous Community. Sodality.

[23] Arham, Sjaf S., Darusman D. (2019). Strategi pembangunan pertanian berkelanjutan di Pedesaan berbasis citra drone. J. Ilmu Lingkung..

[24] W. Wattanacharoensil, S. Talawanich, & L. Jianvittayakit, Multiple qualitative procedures to elicit reverse culture shock experience. MethodsX, 7 (2020) 100766. doi:10.1016/J.MEX.2019.12.007. PMC6992980 32021821

[25] Tjondronegoro S. (1984).

[26] Kolopaking L.M., Tonny F., Hakim L. (2020). Relevansi dan Jejak Pemikiran Prof. Dr. S.M.P. Tjondronegoro dalam Pendidikan Sosiologi Pedesaan. Sodality.

[27] Ault J.K., Spicer A. (2021). Configurational-based institutional analysis: Unbundling the multi-dimensional state fragility construct. MethodsX.

[28] Behrens K., Murata Y. (2021). On quantitative spatial economic models. J. Urban Econ..

[29] Laraswati D., Rahayu S., Pratama A.A., Soraya E., Sahide M.A.K., Maryudi A. (2020). Problem-method fit in forest policy analysis: empirical pre-orientation for selecting tested or innovative social-qualitative methods. MethodsX.

[30] Peraturan Bupati Tapanuli Utara, Data Desa Presisi (2022).

[31] Firnawati K., Sjaf S. (2021). IOP Conf*erence* Ser*ies* Earth Environ*mental* Sci*ence*.

[32] Bell R., Ward P.S., Killilea M.E., Tamal M.E.H. (2016). Real-time social data collection in rural Bangladesh via a “microtasks for micropayments” platform on android smartphones. PLoS ONE.

[33] Zhang S., Wu Q., van Velthoven M.H.M.M.T., Chen L., Car J., Rudan I., Zhang Y., Li Y., Scherpbier R.W. (2012). Smartphone versus pen-and-paper data collection of infant feeding practices in rural China. J. Med. Internet Res..

